# Complex interactions between bacteria and haemosporidia in coinfected hosts: An experiment

**DOI:** 10.1002/ece3.6318

**Published:** 2020-04-29

**Authors:** María Teresa Reinoso‐Pérez, Keila V. Dhondt, Agnes V. Sydenstricker, Dieter Heylen, André A. Dhondt

**Affiliations:** ^1^ Department of Natural Resources Cornell University Ithaca New York; ^2^ Laboratory of Ornithology Cornell University Ithaca New York; ^3^ Department of Microbiology and Immunology College of Veterinary Medicine Cornell University Ithaca New York; ^4^ Department of Integrative Neurosciences Binghamton University Binghamton New York; ^5^ Department of Ecology and Evolutionary Biology Princeton University Princeton New Jersey; ^6^ Interuniversity Institute for Biostatistics and statistical Bioinformatics Hasselt University Diepenbeek Belgium; ^7^ Department of Ecology and Evolutionary Biology Cornell University Ithaca New York

**Keywords:** coinfection, *Haemorhous mexicanus*, house finch, interaction, *Leucocytozoon*, *Mycoplasma gallisepticum*, nonbreeding season, *Plasmodium*

## Abstract

1. Hosts are typically coinfected by multiple parasite species whose interactions might be synergetic or antagonistic, producing unpredictable physiological and pathological impacts on the host. This study shows the interaction between *Plasmodium* spp. and *Leucocytozoon* spp. in birds experimentally infected or not infected with *Mycoplasma gallisepticum*.
2. In 1994, the bacterium *Mycoplasma gallisepticum* jumped from poultry to wild birds in which it caused a major epidemic in North America. Birds infected with *M*. *gallisepticum* show conjunctivitis as well as increased levels of corticosterone.
3. Malaria and other haemosporidia are widespread in birds, and chronic infections become apparent with the detectable presence of the parasite in peripheral blood in response to elevated levels of natural or experimental corticosterone levels.
4. Knowing the immunosuppressive effect of corticosterone on the avian immune system, we tested the hypothesis that chronic infections of *Plasmodium* spp. and *Leucocytozoon* spp. in house finches would respond to experimental inoculation with *M. gallisepticum* as corticosterone levels are known to increase following inoculation.
5. *Plasmodium* spp. infection intensity increased within days of *M. gallisepticum* inoculation as shown both by the appearance of infected erythrocytes and by the increase in the number and the intensity of positive PCR tests.
6. *Leucocytozoon* spp. infection intensity increased when *Plasmodium* spp. infection intensity increased, but not in response to *M. gallisepticum* inoculation. *Leucocytozoon* spp. and *Plasmodium* spp. seemed to compete in the host as shown by a negative correlation between the changes in their PCR score when both pathogens were present in the same individual.
7. Host responses to coinfection with multiple pathogens measured by the hematocrit and white blood cell count depended on the haemosporidian community composition. Host investment in the leukocyte response was higher in the single‐haemosporidia‐infected groups when birds were infected with *M. gallisepticum*.
8. A trade‐off was observed between the immune control of the chronic infection (*Plasmodium* spp.*/Leucocytozoon* spp.) and the immune response to the novel bacterial infection (*M. gallisepticum*).

## Graphical abstract

Hosts are typically coinfected by multiple parasites whose interactions might be synergetic or antagonistic, producing unpredictable impacts on the host. This study shows the interaction between *Plasmodium* spp. and *Leucocytozoon* spp. in birds experimentally infected or not infected with *Mycoplasma gallisepticum*.

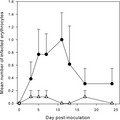

## 1 INTRODUCTION

Hosts are typically coinfected by multiple parasite species (Cox, 2001; Rynkiewicz, Pedersen, & Fenton, 2015). Effects of coinfections on the co‐occurring pathogens vary. Infectious agents can benefit (Diuk‐Wasser, Vannier, & Krause, 2016) or suffer (Ezenwa, Etienne, Luikart, Beja‐Pereira, & Jolles, 2010; Telfer et al., 2010) from the presence of another pathogen in the same host, while some coinfections seem not to affect any of the parasites present (Butcher, 1992).

Studies on the interaction between haemosporidia and other pathogens such as viruses, bacteria, and helminths showed differences in haemosporidian parasitemia between coinfected hosts and control groups (Cox, 2001; Epelboin et al., 2012; Rooth & Bjorkman, 1992). As concerns, coinfections of blood parasites in wild birds both competition and facilitation can influence infection rates (Clark, Wells, Dimitrov, & Clegg, 2016; Meixell et al., 2016). Furthermore, responses of parasites belonging to different genera of haemosporidia to a coinfection might further depend on the pronounced differences in their life cycle as well as on their life stage. Asexual reproduction of *Plasmodium* spp. is erythrocytic (takes thus place inside the red blood cells), while asexual reproduction of *Haemoproteus* spp. and *Leucocytozoon* spp. takes place outside those cells or is thus exoerythrocytic (Valkiūnas, 2005; Valkiūnas & Iezhova, 2017).

A host shift of the bacterium *Mycoplasma gallisepticum* (further *M. gallisepticum*) from poultry to wild birds (Hochachka et al., 2013) caused a major epidemic of mycoplasmal conjunctivitis in house finches *Haemorhous mexicanus* starting around 1994 (Dhondt, Tessaglia, & Slothower, 1998; Fischer, Stallknecht, Luttrell, Dhondt, & Converse, 1997; Ley, Berkhoff, & Levisohn, 1997). *M. gallisepticum* has now spread across most of the United States (Dhondt et al., 2005; Fleming‐Davies et al., 2018), and it has been proven to be an excellent experimental system. As house finches are frequently infected with diverse genera of haemosporidia (Davis, Hood, & Hill, 2013; Dhondt, Dhondt, & Nazeri, 2017; Kimura, Dhondt, & Lovette, 2006), coinfections of *M. gallisepticum* and haemosporidian species must be frequent in wild house finches which could impact both parasites (Dhondt & Dobson, 2017).

*Plasmodium* infection intensity increases when corticosterone levels naturally increase in the breeding season but also when they are experimentally increased (Applegate, 1970). Thus, in house sparrows, *Passer domesticus* with a latent infection of *Plasmodium relictum,* a daily injection with corticosterone during a 10‐day period in winter caused a relapse of the *P. relictum* infection resulting in detectable parasites in blood smears, while in control birds the parasites could not be found in erythrocytes. The natural increase in corticosterone levels at the onset of the breeding season also caused this relapse although corticosterone injections accelerated it resulting in a higher infection intensity (Applegate, 1970). Schoenle et al. (2019) similarly showed that exogenous glucocorticoids amplified *Plasmodium* spp. burden but less so in red‐winged blackbirds *Agelaius phoeniceus* coinfected with *Leucocytozoon* spp. and/or* Haemoproteus* spp. By combining these results with the observation that in house finches corticosterone levels increase following an experimental infection with *M. gallisepticum* (Love, Foltz, Adelman, Moore, & Hawley, 2016), we can hypothesize that in birds chronically infected with *Plasmodium* spp., and possibly with other haemosporidia, parasitemia would increase following a *M. gallisepticum* infection (Dhondt & Dobson, 2017). The primary objective of this study, therefore, was to test this hypothesis and determine whether infection intensity of haemosporidian parasites increases following an experimental infection of house finches with *M. gallisepticum.* A secondary question was whether *Plasmodium* spp. and *Leucocytozoon* spp. would respond in similar ways.

## 2 METHODS

### 2.1 Birds: capture, testing, and housing

Thirty‐two juvenile house finches were captured between the end of May and August 2017 in Ithaca, Tompkins County, New York (46.460547 N, −76.465969 W), under permit (New York State Fish and Wildlife License 39, Albany, NY; United States Geological Survey, Department of the Interior, Laurel, MD, permit 22669). Birds were kept in individual wire bar cages (45 × 45 × 75 cm) until September 2017 when the experiment started. The cages were placed in aviaries inside a large closed barn (Dhondt, States, Dhondt, & Schat, 2012) and therefore inaccessible to mosquito vectors. In all cages, the arrangement of perches, water, and food containers was identical. Water and food (Roudybush, Inc. Cameron Park, CA (2/3) mixed with sunflower seeds (1/3)) were offered ad libitum. All experiments were approved by Cornell University's IACUC protocol 2009‐0034. Before starting the experiment, all birds were negative for *M. gallisepticum* by three different methods: visual inspection for eye lesions (Sydenstricker et al., 2006), real‐time polymerase chain reaction (qPCR) designed to test for the presence of the bacteria using the DNA from conjunctival swabs (Grodio, Dhondt, O'Connell, & Schat, 2008), and rapid plate agglutination (RPA) to test for the presence of *M. gallisepticum*‐specific antibodies in blood (Sydenstricker et al., 2006).

### 2.2 Testing for the presence of haemosporidia

**FIGURE 1:**
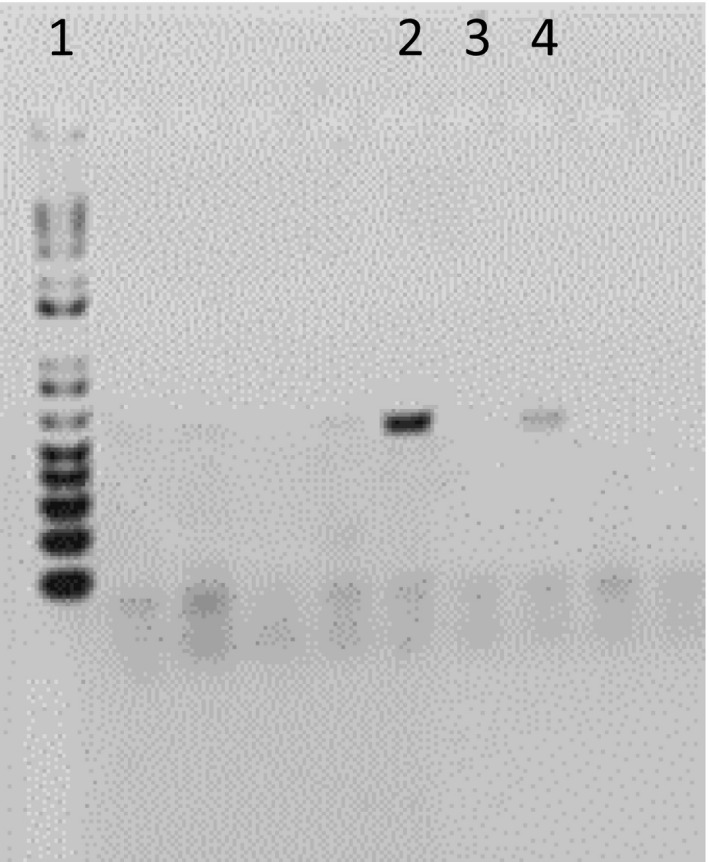
Output of agarose gel electrophoresis of the mitochondrial cytochrome *b* gene from parasites belonging to three different genera of haemosporidia, *Plasmodium*, *Haemoproteus,* and *Leucocytozoon*. Lane 1: 1kb plus ladder, lane 2: a clear band with score 2, lane 3: no band or amplification with score 0, and lane 4: a faint band with score 1

The presence of haemosporidian parasites was tested in all birds, and the parasite lineage was identified when possible. Blood samples were taken at the same time every sampling day starting at 9 a.m. with the same bird and sampling the birds in the same sequence. DNA was extracted from each blood sample using the DNeasy Blood & Tissue Isolation Kit (Qiagen) according to the manufacturer's instructions. To verify DNA integrity, extractions were screened on 2% agarose gels. For the molecular diagnosis, the protocol for nested polymerase chain reaction (PCR) described by Bensch, Hellgren, and Pérez‐Tris (2009) was followed using the primers described in Bensch et al. (2000); Hellgren, Waldeström, and Bensch (2004); Waldenström, Bensch, Hasselquist, and Östman (2004). It targets the mitochondrial cytochrome *b* gene in the haemosporidian genome. For each sample, separate reactions of nested PCR were performed to detect *Plasmodium* spp./*Haemoproteus* spp. and *Leucocytozoon* spp. For *Plasmodium* spp./*Haemoproteus* spp. PCRs, two pairs of specific primers were used (HAEMNF CATATATTAAGAGAATTATGGAG‐HAEMNR2 AGAGGTGTAGCATATCTATCTAC and HAEMF ATGGTGCTTTCGATATATGCATG‐HAEMR2 GCATTATCTGGATGTGATAATGGT). For *Leucocytozoon* spp., two pairs of specific primers were used (HAEMNFI CATATATTAAGAGAAITATGGAG‐HAEMNR3 ATAGAAAGATAAGAAATACCATTC and HAEMFL ATGGTGTTTTAGATACTTACATT‐HAEMR2L CATTATCTGGATGAGATAATGGIGC). The PCR was repeated three times for each sample. Given the variation among replicates in band intensity, the bands were visually scored on a scale of 0–2. All PCR products were confirmed by 2% agarose gel electrophoresis and visualized with ethidium bromide staining. Photographs of each gel were taken using a UV transilluminator with Kodak Gel Logic Digital Imaging System. All photographs were printed on the same laser printer and used to score all PCR products. Samples with the absence of a band were scored as 0; a faint band was scored as 1; and a strong band was scored as 2 (see Figure 1). For each individual, we summed the three scores to calculate a PCR score to reflect the intensity of infection. The scoring was done blind and independently by MRP and AAD. Due to our small sample size, we analyzed the PCR bands in two ways. First, we counted bands (standard method). Second, we employed the band intensity method described above. All PCR products for any haemosporidian species were sequenced and compared to the MalAvi database (Bensch et al., 2009).

Three thin blood smears were prepared per individual bird following standard techniques for haemosporidian studies (Valkiūnas, 2005) using an aliquot taken from the brachial vein. Blood smears were air‐dried, fixed with 100% methanol, and immediately stained with Giemsa stain prepared as per Petithory, Ardoin, and Ash (2005). The slides were examined using a Meiji Techno MT 4000 Biological Microscope using an oil immersion objective (100×). Parasitemia was quantified for each smear by recording the number of infected cells in 100 random fields (Godfrey, Fedynich, & Pence, 1987) that each had approximately 200 erythrocytes for a total of 20,000 erythrocytes (red blood cells, RBCs).

The infection intensity was scored by using the number of infected RBC and by using the PCR score. The hypothesized impact of the *M. gallisepticum* inoculation on haemosporidian infection intensity was determined using both changes in the PCR score and changes in the number of infected RBC between day 0 (preinfection score) and day 24 postinfection (PI) and comparing birds infected with *M. gallisepticum* and the control group.

### 2.3 Inoculation with *M. gallisepticum*

Roughly half of the birds were inoculated with *M. gallisepticum* (further: experimental birds), while the others were inoculated with Frey's medium, the standard medium in which to grow these bacteria (Kleven,^2008^ ) (further: controls or control birds). Given that before the inoculation, some birds were infected with *Plasmodium* spp., some with *Leucocytozoon* spp., some with neither, and some with both we assigned about half of the birds in each of the groups to each treatment*.* Thus, among the birds with only *Plasmodium* spp. 12 were inoculated with *M. gallisepticum*, and 10 kept as controls; among the eight birds in which only *Leucocytozoon* spp. was detected four individuals were and four were not inoculated with *M. gallisepticum.* The remaining two birds (no haemosporidia or both genera detected) were also inoculated with *M. gallisepticum*. In total, 18 house finches were inoculated with *M. gallisepticum* and 14 birds were kept as controls.

Each bird in the experimental group was inoculated on 15 September 2017 (day 0) with 50 µl of the *M. gallisepticum* isolate CA2015.022‐3(2P) at 2.8 × 10^7^ CFU/ml in each eye (Ley, Hawley, Geary, & Dhondt, 2016). The birds in the control group were similarly inoculated with Frey's medium.

### 2.4 Procedures following *M. gallisepticum* infection

To measure the host response to *M. gallisepticum* infection, the eye lesions of each eye were scored in all birds on a scale of 0 (no lesions) to 3 (severe lesions) following Sydenstricker et al. (2006) on days 0, 3, 5, 7, 11, 13, 15, 17, 20, and 24 PI. The presence of *M. gallisepticum*‐specific antibodies was tested on days 13 and 24 PI (Sydenstricker et al., 2006).

On days 0, 3, 5, 7, 11, 13, 15, 17, 20, and 24 PI, three thin blood smears were made to count the number of infected erythrocytes and compare parasitemia preinfection (day 0) and postinfection. To measure changes in the intensity of haemosporidian infection following *M. gallisepticum* infection changes in *Plasmodium* spp. and *Leucocytozoon* spp., PCR score of each bird between day zero and day 24 PI was calculated.

Hematocrit (Hct) was measured on days 0, 3, 5, 7, 11, 13, 15, 17, 20, and 24 PI. To estimate the number of white blood cells (WBCs) per ml, WBCs were counted in 10 fields at 40× and the total number was multiplied by 200 following Fudge (2000).

### 2.5 Statistical analysis

The statistical analyses were conducted using Statistix10 (Analytical Software) and SAS v 9.3 (SAS Institute). Graphs were made using SigmaPlot 11. When distributions of residuals were not following the exponential family of probability distributions (normal, Poisson, binomial, or negative binomial), nonparametric tests were used for comparison of means between two (Mann–Whitney *U* test) or more (Kruskal–Wallis *H* test) groups. Fisher's exact tests were used to compare frequencies because of small sample sizes, and Pearson's correlation coefficients were used to calculate correlations between the PCR scores on day 0 and the change in PCR score following *M. gallisepticum* inoculation to test for possible “regression to the mean” effects. Impacts of inoculation on *Plasmodium* spp. parasite loads in individual birds were tested via generalized estimating equations (Levin et al., 2013) for negative binomial distributed residuals, taking into account the dependence of repeated observations over several days within the same individual using the exchangeable working correlation matrix (Molenberghs & Verbeke, 2005). Repeated measures ANOVAs were used for comparisons between day 24 and day 0, when residuals were normally distributed. For the explicit analyses of longitudinal profiles of hematocrit levels (normally distributed) and white blood cell counts (Poisson‐distributed), GEEs were fitted allowing for dependency of repeated measurements over time within the same bird (Molenberghs & Verbeke, 2005). The models test whether the changes in health measures over time are related to the *M. gallisepticum* inoculation and the presence of haemosporidian parasites. The mean structure of the profiles was modeled according to a piecewise regression model, which explicitly models the bird's measurements until, and after day 7:

$$\text{Health}\;\text{measure}=\alpha_{0}+\left[\left(1-x\right)\text{Time}\;+Qx\right]\beta_{0}+x\left(\text{Time}-Q\right)\beta_{1}$$

with *x* = 0 (until day 7), *x* = 1 (after day 7), and *Q* = day 7 (i.e., the moment of flexion) (Verbeke & Molenberghs, 2001). Parameters *α*_0_, *β*_0_, and *β*_1_ were modeled in relation to the following fixed effects: *M. gallisepticum* treatment (yes/no), and haemosporidian presence (yes/no), for each of the following groups: *Plasmodium* spp., *Leucocytozoon* spp., and both. Intercepts (*α*_0_) for each combination of parasites were left in the model, to allow the profiles to begin as close as possible to their empirical mean (day 0). Effects of parasites on the changes with time (*β*s) were evaluated via a stepwise selection procedure: The model was iteratively refitted after exclusion of the least significant fixed effect based on type 3 tests (See Appendix), until only significant main effects on the changes with time (*β*s) and their lower‐order interaction terms were left. *α* = .05 was chosen as the lowest acceptable level of significance. We used the identity link for variables with normally distributed residuals (hematocrit), while for the Poisson‐distributed residuals in the leukocyte counts we used the log link. The piecewise model explained the data better than a curvilinear model with day and day^2^. The backward selection using two different intervals (*β*_0_ from 0 to 6.9, or 0 to 7.1) generated almost identical results (only the latter will be reported).

## 3 RESULTS

### 3.1 Eye lesions in response to *M. gallisepticum* inoculation

**FIGURE 2:**
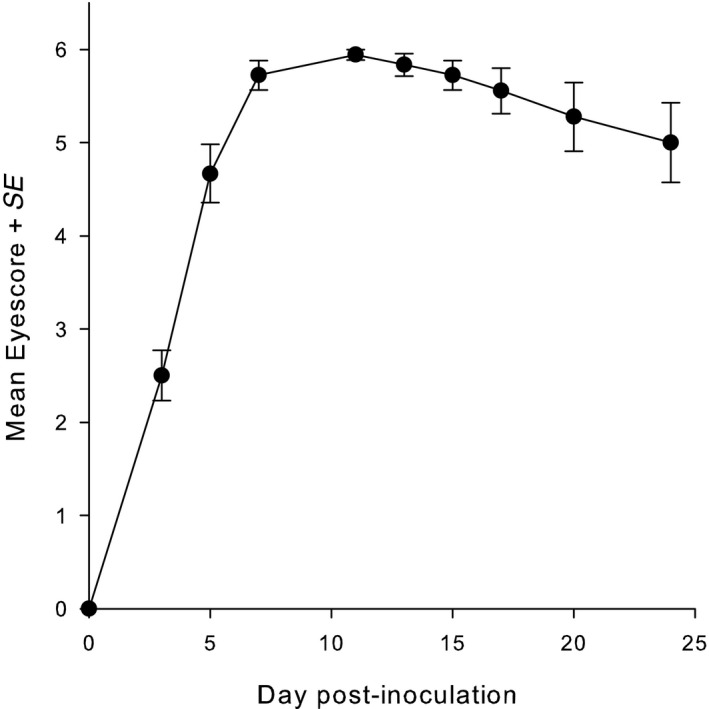
Changes in eye score from day 0 to day 24 PI (mean ± *SE*) in house finches infected with *Mycoplasma gallisepticum* CA2015. The maximum eye score is 6

Following *M. gallisepticum* inoculation, all house finches developed conjunctivitis in both eyes by day 3 PI. Eye scores reached a maximum by day 11 PI, and birds maintained severe signs of disease to day 24 PI (see Figure 2). In all birds in this group, *M. gallisepticum*‐specific antibodies were detected on day 13 and day 24. None of the control birds developed lesions, and none had antibodies (Fisher's exact test: *p* < .0001). The mean eye score did not differ between birds infected with *Plasmodium* spp.*, Leucocytozoon* spp., and both (Kruskal–Wallis ANOVA *H* = 5.46, *df* = 2; *p* = .07).

### 3.2 Haemosporidian occurrence in the peripheral blood before and after inoculation

Before the 32 birds were inoculated with *M. gallisepticum* or with control medium, our triplicated PCR tests found 23 house finches to be naturally infected with a *Plasmodium* lineage. Seven of them were infected with PADOM11, three with WW3, and in the other 13 we did not get enough good quality DNA to be able to assign or identify the parasite lineage. Ten house finches were infected with a *Leucocytozoon* lineage (9 CB1, 1 CARFLA04). Two of these house finches were coinfected by *Plasmodium* (PADOM11) and *Leucocytozoon* (CB1). Out of the 32 birds, only one individual was negative for all PCR test. We did not get any sequences by PCR from infected birds that belonged to the genus *Haemoproteus.* In none of the birds did we detect any infected blood cells.

The number of positive PCR tests for *Plasmodium* spp. in replicated samples of the same individual varied between 1 (*n* = 4) and 3 (*n* = 9) and the summed PCR scores in birds that tested positive for at least one PCR test varied between 1 and 5.5 (mean 2.78 ± *SE* 0.247). For *Leucocytozoon* spp., all three tests in each bird were either positive or negative and the PCR score varied between 2.75 and 6 (4.83 ± *SE* 0.379).

On day 24 PI *Plasmodium* spp. was detected in the same individuals as before the start of the experiment, but *Leucocytozoon* was detected in 10 additional birds whereby two additional haplotypes (CARCHL04 and CNEORN01) were identified. Half of those additional birds were in the control group, suggesting that handling stress and captivity may have played a role in *Leucocytozoon*'s emergence*.* All 10 additional *Leucocytozoon*‐infected birds were coinfected with *Plasmodium* spp., bringing the total number of birds coinfected with different haemosporidian genera to 12.

Both the band counting and the band intensity methods revealed qualitatively similar responses to *M. gallisepticum* inoculation (Table 1). For house finches infected with *Plasmodium* spp., both methods showed a significant difference in the increase in haemosporidian infection intensity from day 0 to day 24 between birds inoculated with *M. gallisepticum* and the control birds, while for birds infected with *Leucocytozoon* spp. neither comparison showed a significant change.

**TABLE 1:** Comparison of change in haemosporidian infection detection from day 0 to day 24 between birds inoculated with *Mycoplasma gallisepticum* and the control birds using, on the one hand, the number of positive bands in triplicated tests (band count), and on the other hand, a PCR score that combines the number of bands and band intensity (band score)

Haemosporidia	MG_treatment	Sample size	Band_score		Band_count	
			Mean	*SE*	Mean	*SE*
*Plasmodium*	MG +	18	1.458	0.343	0.722	0.165
	MG −	14	−0.411	0.197	−0.214	0.213
	*t*_(30)_		4.375		4.16	
	*p*‐value		<.0001		<.0001	
*Leucocytozoon*	MG +	18	0.5	0.347	0.444	0.446
	MG −	14	−0.232	0.412	0.214	0.423
	*t*_(30)_		1.367		0.355	
	*p*‐value		.18		.12	

Note

The two methods generated qualitatively similar results as shown by the similar *p*‐values of the *t* test.

### 3.3 Quantitative responses of *Plasmodium* spp. and *Leucocytozoon* spp. in the peripheral blood to *M. gallisepticum* coinfection

**FIGURE 3:**
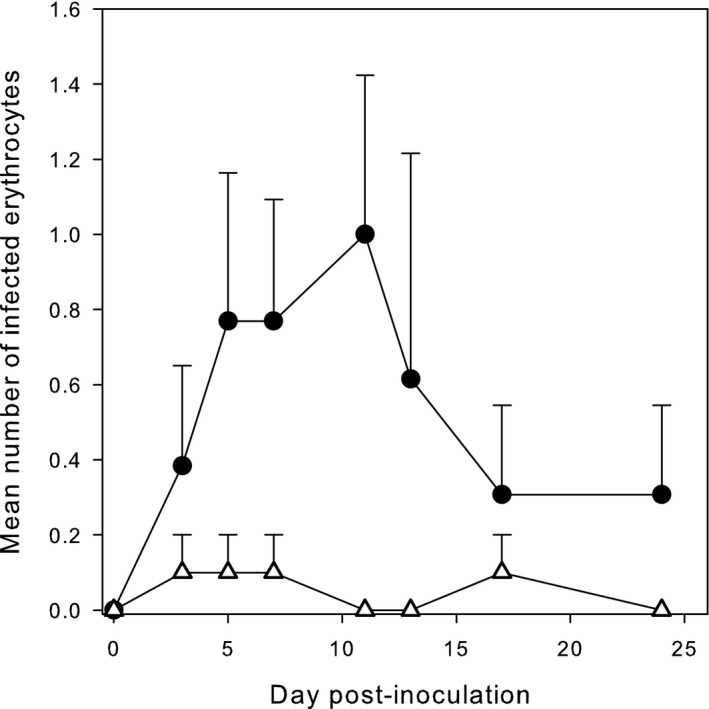
Changes in number (mean ± *SE*) of infected erythrocytes per 20,000 cells counted in house finches infected with *Mycoplasma gallisepticum* (circles) or not (triangles) from day 0 to day 24 PI

In the experimental birds, the average number of red blood cells (RBCs) containing *Plasmodium* spp., that was zero before the inoculation, had increased by day 3 PI, continued to increase to day 5, reaching a maximum by day 13 (0.92 ± *SE* 0.65 per 20,000 RBCs) after which it decreased again (Figure 3). Among the 23 birds proven to be *Plasmodium* spp.‐positive by PCR, infected RBCs were observed more frequently in the experimental birds (9 of 13) than in the control birds (1 in 10) (two‐tailed Fisher's exact test: *p* = .0097). The maximum number of infected RBC in any individual was 8 per 20,000 RBC on day 13 PI. From the smears of the control birds, *Plasmodium*‐infected RBCs were detected in one single bird: 1 per 20,000 cells on days 3, 5, 7, and 17 PI. The GEE using individual erythrocyte counts and controlling for day shows a significantly larger number of infected erythrocytes in birds inoculated with *M. gallisepticum* than in control birds (inoculated versus controls: 2.37 ± *SE* 1.03, log link; *Z* = −2.30, *df* = 1, *p* = .02).

**FIGURE 4:**
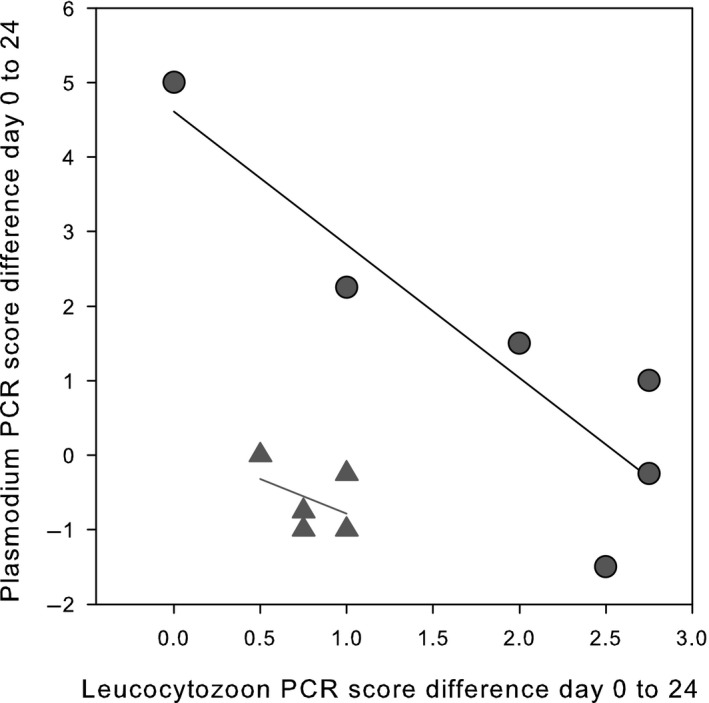
Change in *Leucocytozoon* PCR score plotted against the change in *Plasmodium* PCR score from day 0 to day 24 PI in the presence (circles) or absence (triangles) of *Mycoplasma gallisepticum.* All birds were coinfected with parasites belonging to two different genera of haemosporidia

In line with the above effects of *M. gallisepticum* infection on cytological presence of *Plasmodium* spp., in the peripheral blood the change in PCR scores from day 0 to day 24 was significantly higher in experimental than in control birds (see Figure 4) (Repeated measures ANOVA *F*_1,21_ = 16.50, *p* = .0006). Using only the subset of birds in which *Leucocytozoon* spp. had not been detected (*N* = 12 birds), a repeated measures ANOVA still showed a very significant effect of *M. gallisepticum* on *Plasmodium* spp. (*F*_1,9_ = 15.02, *p* = .004; control birds *n* = 5, mean −0.55 ± 0.472; experimental birds *n* = 7, mean 2.11 ± 0.399). In contrast, *Leucocytozoon* PCR score did not increase significantly following *M. gallisepticum* inoculation (repeated measures ANOVA *F*_1,18_ = 1.92, *p* = .18). No infected RBCs were found in birds infected with *Leucocytozoon* spp. alone.

### 3.4 Effects of haemosporidian coinfection

Possible effects of coinfection of parasites belonging to these two genera of haemosporidia on the response to *M. gallisepticum* inoculation were tested in two ways*.* First by performing a repeated measure ANOVA to detect a possible effect of the presence of the other haemosporidia genus in combination with *M. gallisepticum* on changes in the PCR score between day 0 and day 24. Thus, the presence/absence of *Leucocytozoon* did not result in a significant difference in a change in *Plasmodium* PCR score between experimental and control birds (*F*_1,19_ = 0.17, *p* = .68; Figure 4). In contrast, in birds in which *Plasmodium* spp. *w*as present, the change in *Leucocytozoon* spp. PCR score (*n* = 11, change + 1.98) was much larger than in birds without *Plasmodium* spp. (*n* = 9, change = −1.10) a very significant difference (*F*_1,17_ = 13.58, *p* = .002). In this analysis, neither the interaction term (*F*_1,16_ = 0.08, *p* = .78) nor the direct effect of *M. gallisepticum* (*F*_1,16_ = 3.40, *p* = .09) was statistically significant. The change in *Leucocytozoon* PCR score from day 0 to day 24 PI following *M. gallisepticum* inoculation in birds in which *Plasmodium* spp. had not been detected did not differ between experimental (*n* = 4, mean −0.4) and control birds (*n* = 5, mean −1.81) (repeated measures ANOVA *F*_1,17_ = 1.06, *p* = .34).

In a second analysis, only birds found to be infected with parasites belonging to both genera of haemosporidia were included (*N* = 11 birds). We then tested for an effect of *M. gallisepticum* on change in *Plasmodium* PCR score while including the *Leucocytozoon* spp. change in PCR score as a covariate. A two‐way ANOVA shows a very significant effect of *M. gallisepticum *(*F*_1,8_ = 35.86, *p* = .0003), and a very significant effect of *Leucocytozoo*n (*F*_1,8_ = 26.98, *p* = .0008). The negative coefficient (−1.77 ± *SE* 0.34) indicates that the changes in PCR scores of the two genera of haemosporidia from day zero to day 24 PI are inversely related (see Figure 4). This result is not caused by a “regression to the mean” effect as (a) the PCR scores on day 0 are not negatively correlated to the change in PCR score between day 0 and day 24 in either genus (*Leucocytozoon*: *n* = 11. *r* = −0.50, *p* = .12; *Plasmodium*: *n* = 11. *r* = −0.43, *p* = .19), and (b) there was no negative correlation between the PCR score on day 0 of *Leucocytozoon* and *Plasmodium* (*n* = 11. *r* = −0.25, *p* = .46).

### 3.5 White blood cell counts

**FIGURE 5:**
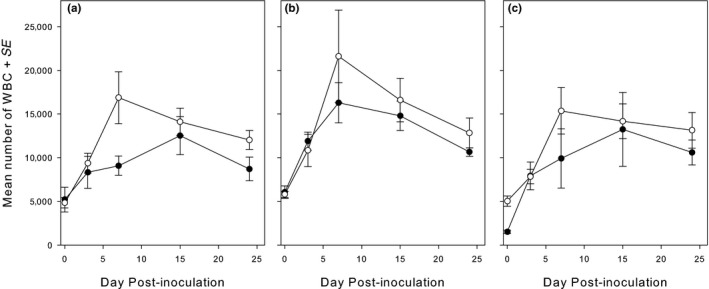
Variation in number of white blood cells (WBCs) (mean ± *SE*) following inoculation with *Mycoplasma gallisepticum*. *Open circles,* experimental birds; *filled circles,* control birds. (a) birds infected with *Plasmodium* only; (b) bird infected with *Leucocytozoon* only; and (c) birds coinfected with both *Plasmodium and Leucocytozoon*. During the first 7 days, there were no differences between the groups, while later the effect of *M. gallisepticum on* the change in WBC was significantly less than in the other birds

The temporal changes in white blood cell counts until day 7 PI were affected by an interaction between *M. gallisepticum* and haemosporidian coinfections as shown by the significant three‐way interaction Period_1*MG*Haem (*χ* ^2^ = 6.88, *df* = 2; *p* = .032; Table 2). Host investment in the leukocyte response was higher in the single‐haemosporidia‐infected groups with *M. gallisepticum*, as shown by the parameter estimates for the *Plasmodium*‐infected group (difference contr.‐exp.: −0.075 ± *SE* 0.037, *Z* = −2.01; *p* = .044) and the *Leucocytozoon*‐infected group (difference contr.‐exp.: −0.045 ± *SE* 0.022, *Z* = −2.03; *p* = .043) in Figure 5. However, neither in the control birds nor in the experimental birds in the double‐haemosporidia‐infected group, was there a significant change over time (difference contr.‐exp.: 0.028 ± *SE* 0.017, *Z* = 1.64; *p* = .10). After the strong increase in the single‐haemosporidia‐infected groups (day 0–day 7 PI), the decrease from day 7PI onwards depended on the presence of haemosporidia but not on the *M. gallisepticum* infection (two‐way interaction period_2*Haem; *χ* ^2^ = 6.98, *df* = 2; *p* = .031; Table 2). The decrease tended to be slightly stronger in the *Leucocytozoon* spp.‐infected group than in the *Plasmodium* spp.‐infected group (difference: 0.014 ± *SE* 0.008; *Z* = 1.83; *p* = .068), although not statistically significant in the double‐haemosporidia‐infected birds (See Appendix).

**TABLE 2:** Score statistics of type 3 tests before exclusion from the model when *p* ≥ .05

	*df*	WBC count		Hematocrit		Parameter
		χ^2^	*p*	χ^2^	*p*	
MG‐bacteria	**1**	**0.05**	**.82**	**0.22**	**.64**	*α*_0_
Haemosporidian	**2**	**9.6**	**.0082**	**3.32**	**.19**	
MG*Haemosporidia	**2**	**2.27**	**.32**	**6.12**	**.047**	
Period_1	**1**	**23.48**	**<.0001**	**11.7**	**.0006**	*β*_0_
Period_1*MG‐bacteria	**1**	**2.92**	**.087**	1.85	.17	
Period_1*Haemosporidia	**2**	**0.4**	**.82**	0.4	.82	
Period_1*MG‐bacteria*Haemosporidia	**2**	**6.88**	**.032**	2.82	.24	
Period_2	**1**	**12.79**	**.0003**	**13.47**	**.0002**	β_1_
Period_2*MG‐bacteria	1	1.87	.17	**4.47**	**.035**	
Period_2* Haemosporidia	**2**	**6.98**	**.031**	**7.77**	**.021**	
Period_2* MG‐bacteria*Haemosporidia	2	0.8	.67	2.51	.28	

Note

In bold and underlined: factors left in the model. Only significant main effects on the changes with time (*β*s) and their lower‐order interactions were left in the model. Intercepts (*α*_0_) for each combination of parasites were left in the model to allow the profiles to begin as close as possible to their empirical mean (day 0). Parameter estimates are discussed in the main text, and a detailed overview is given in the Appendix.

Period1 = (1 − x) × (day) + *Q* × *x*; Period2 = *x* × (day − Q).

*x* = 0 (before manipulation); *x* = 1 (after manipulation); *Q* = day 7.

*α*_0_: intercept_,_ *β*_0_: slope of the profile curve before flexion (day 7 included)_,_ *β*_1_: slope of the profile after day 7.

Factor “MG‐bacteria” has two levels (noninfected and *M. gallisepticum*‐infected); “Haemosporidia” has three levels (*Plasmodium, Leucocytozoon*, and both).

### 3.6 Hematocrit

**FIGURE 6:**
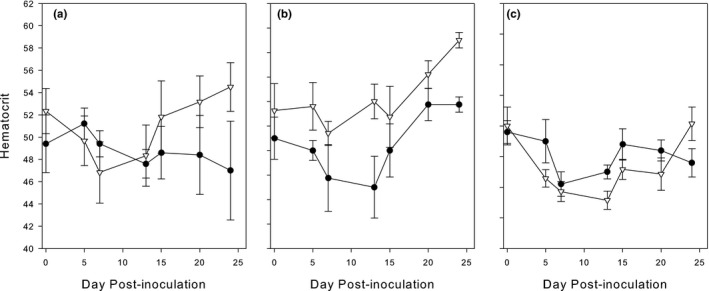
Variation in hematocrit values (mean ± *SE*) in house finches following inoculation with *Mycoplasma gallisepticum. Open triangles,* experimental birds; *filled circles,* control birds. (a) *Plasmodium* alone; (b) *Leucocytozoon* alone; and (c) both *Leucocytozoon* and *Plasmodium*

We were interested whether following inoculation with *M. gallisepticum* hematocrit values would change and whether changes would vary with the presence of haemosporidian parasites. The hematocrit values fluctuated during the experiment but remained within the normal range. Seven to ten days after the *M. gallisepticum* infection, hematocrit levels reached their lowest level, the rate of decrease being similar between groups (Figure 6). The rate of hematocrit recovery, however, significantly differed among treatment groups (Interactions Period_2*MG; *χ* ^2^ = 4.47, *df* = 1; *p* = .031, and Period_2* Haem *χ* ^2^ = 7.77, *df* = 2; *p* = .021; Table 2). The control birds recovered more slowly than the experimental birds (diff. control – exp.: −0.20 *SE* 0.08; *Z* = −2.31; *p* = .021). Furthermore, the recovery rate in the *Leucocytozoon*‐infected birds was faster than in the birds infected by both genera of haemosporidia (difference −0.27 *SE* 0.07; *Z* = −3.68; *p* = .0002; Appendix), but not significantly different from the birds infected with *Plasmodium* only (difference −0.08 *SE* 0.11; *Z* = −0.73; *p* = .46).

## 4 DISCUSSION

The primary objective of our experiment was to determine the extent to which the infection intensity of birds chronically infected with haemosporidian parasites would increase if they were also infected with the bacterium *M. gallisepticum,* and explore, as a secondary question, the extent to which *Plasmodium* spp. and *Leucocytozoon* spp. would respond in similar ways. The hypothesis stemmed from earlier experimental results showing that *Plasmodium* spp. infection intensity increased following injections with corticosterone (Applegate, 1970) [recently confirmed by (Schoenle et al., 2019)], and recent experimental results documenting that house finch corticosterone levels naturally increased following a *M. gallisepticum* infection (Love et al., 2016). As hypothesized by Dhondt and Dobson (2017), we did observe a parasitemia increment in birds chronically infected with *Plasmodium* sp. following a *M. gallisepticum* infection. As *Leucocytozoon* spp. is also more prevalent during the breeding season when corticosterone levels naturally increase in birds (Applegate, 1970), we expected a similar response of *Leucocytozoon* spp. However, because Schoenle et al. (2019) showed that the *Plasmodium* spp. burden increased less when the birds were coinfected with *Leucocytozoon* spp., and not at all when coinfected with *Haemoproteus* spp. we considered the possibility of a within‐host interaction between *Leucocytozoon* spp. and *Plasmodium* spp. Our birds were not infected with *Haemoproteus* spp.

### 4.1 Methodological issues

Even though PCR tests have the potential to detect DNA of pathogens at very low parasitemia, the technique presents some technical challenges: The DNA may suffer damages from sampling, through transportation, storage, and extraction; the quality and purity of the material determines the results of the technique. Further, given that birds have nucleated red blood cells, the total DNA in a sample contains only low copy numbers of the parasite's target sequence against a high background level of nontarget host DNA (Freed & Cann, 2006). Variation in detection might be the result of these factors. In this study, we could not determine the *Plasmodium* linage recovered from 13 birds. These correspond to the samples with a very low values in the PCR score after triplicate molecular diagnosis. Moreover, those birds also remained with a very low parasitemia when we examined their blood smears after the *M. gallisepticum* infection. It is very likely that the failure to identify the *Plasmodium* lineage infecting those individuals is the result of low‐quality amplicons due to a very low parasitemia. Although we sequenced every amplicon we obtained from triplicate PCR, no sequences could be identified. On the other hand, every sample was tested separately for *Plasmodium* and *Leucocytozoon*. We were therefore always able to detect coinfection of these two genera.

The usefulness of repeating each PCR test three times was confirmed by differences in parasite DNA detection between replicates that are likely the result of the low parasitemia in birds in which we did not detect parasites by microscopy. The value of this approach is reinforced by the fact that following the inoculation of the birds with *M. gallisepticum,* it was possible to observe an increase in infection intensity for both *Plasmodium* and *Leucocytozoon*, or an increased in the probability to detect an infection (for *Leucocytozoon*).

Because following *M. gallisepticum* inoculation haemosporidian detections using PCR increased, and although our sample size is small, it is possible to evaluate the extent to which triplicate PCR tests still miss infections. To do this, we assume that birds that are PCR‐positive at least once are true positives and that birds in which all six PCR tests (three on day 0 and three on day 24) were negative are true negatives. On day zero and on day 24 PI, all 23 birds with a *Plasmodium* spp. infection were positive at least once. Triplicate test thus do not seem to generate false negatives for *Plasmodium* spp. The *Plasmodium* lineages identified on day 0 and on day 24 were the same. As concerns *Leucocytozoon* spp. *c*ombining the analyses of day zero and day 24 PI, 18 birds were positive at least once by PCR. On day zero, however, only 8 of these birds were positive by PCR. On day 24 PI, this pathogen was detected in 10 additional birds, implying that on day zero *Leucocytozoon* spp. was detected in only 44% of the birds in which we can assume it was present. On day 24 PI, *Leucocytozoon* spp. was detected in 17/18 birds, that is, still not in all the birds where we can assume it was present. For *Leucocytozoon,* we detected different lineages in the same individual in samples from different days showing evidence of coinfections of different *Leucocytozoon* lineages, which made the identification of the lineages in some individuals difficult.

The implication of this is that researchers who test bird blood for haemosporidian parasites in the nonbreeding season are likely to underestimate parasite prevalence, especially if they run the PCR test only once. The problem is further complicated by the fact that we lumped all isolates per genus because our sample size was small and it was therefore not possible to evaluate the extent to which different isolates vary in detection rate by PCR or respond differently to *M. gallisepticum* infection.

Molecular diagnosis suggests that haemosporidian prevalence of house finches around Ithaca, New York, is 100%, a result we only found because of the response of the birds to coinfection with *M. gallisepticum* and repeated testing of the same individuals.

### 4.2 Effect of *M. gallisepticum* on haemosporidian load

Previous studies have shown a variety of reactions on co‐occurring pathogens. Our results showed very different responses to coinfection with *M. gallisepticum* between the two haemosporidian genera. *Plasmodium spp*. parasitemia was higher in birds coinfected with the bacterium, a result consistent with other studies in which malaria parasites and bacteria co‐occur in an individual host (Rooth & Bjorkman, 1992). Our experiment clearly confirmed the hypothesis that *M. gallisepticum* infection leads to an increase in *Plasmodium* spp. infection intensity as both the number of infected erythrocytes and the number and intensity of PCR bands increased in the experimental birds as compared to the controls. Although the increase in the number of *Plasmodium* spp.‐infected RBC was minimal, it was very similar to that obtained by Applegate (1970). When in the middle of winter Applegate‐injected corticosterone into house sparrows that had a latent P*lasmodium relictum* infection 5 of 6 house sparrows relapsed, a proportion similar to 9/13 house finches in our experiment (one‐tailed Fisher's exact test *p* = .48). Although the mean parasite loads in the house finches' RBC were continuously low (<0.92 cells per 20,000 RBC), it was very similar to Applegate's maximum mean number of infected RBC (<2 infected cells/40,000 RBC). Similarly, the highest number of infected RBC in any of his counts was 8/40,000 RBC compared with our 8/20,000 RBC. The similarity between Applegate's results and our results suggests that the putative effect of an increase in corticosterone caused by the *M. gallisepticum* inoculation (Love et al., 2016) was most likely the cause for the relapse of *Plasmodium* spp. in our house finches. This conclusion is further strengthened by the recent work of Santiago‐Alarcon, Carbó‐Ramírez, Macgregor‐Fors, Chávez‐Zichinelli, and Yeh (2018) in which they compared haemosporidian prevalence and infection intensity in house sparrows before and after a 15‐day stress trial. They found a significant increase in infection intensity in the nonurban house sparrows (but not in urban birds) and explained that by suggesting that urban birds are continuously exposed to stress, and hence do not suffer as much from captivity.

The change in number of infected RBC in house finches inoculated with *M. gallisepticum* coincided with an increase in the *Plasmodium* sp. PCR score obtained through our triplicated PCR tests in which band intensity was scored. Performing the PCR test in triplicate and using separate reactions for different genera of haemosporidia do allow one to semi‐quantify the amount of circulating *Plasmodium* spp. and *Leucocytozoon* spp. DNA when levels are very low.

While *Plasmodium* infection intensity increased following *M. gallisepticum* inoculation, there was no additional effect of the latent *Leucocytozoon* infection. The change in *Leucocytozoon* infection intensity following *M. gallisepticum* inoculation, on the other hand, was not caused by the *M. gallisepticum* inoculation itself—as in the absence of *Plasmodium* spp. coinfection there was no significant change in PCR score—but indirectly the result of coinfection with *Plasmodium* spp.

The three pathogens in this study showed complex three‐way interactions and contrasting effects on health‐related impacts on the bird. We documented a direct effect of *M. gallisepticum* on *Plasmodium* spp. infection intensity (earlier we also showed an inverse effect: see Dhondt et al., 2017). The effect of *M. gallisepticum* on *Leucocytozoon* spp. is primarily indirect via the effect on *Plasmodium* spp. *as* no significant difference was found in *Leucocytozoon* spp. burden following a *M. gallisepticum* inoculation when comparing birds in which *Plasmodium* spp. was detected or not. In house finches coinfected with both *Plasmodium* spp. and *Leucocytozoon* spp., the quantitative change in PCR score following *M. gallisepticum* inoculation was very significantly inversely related: The smaller the increase in *Leucocytozoon* PCR score, the larger the increase in *Plasmodium* spp. PCR score. This inverse response of *Plasmodium* spp. and *Leucocytozoon* spp. following *M. gallisepticum* inoculation suggests the existence of within‐host interspecific competition between these two genera of haemosporidia impacting infection dynamics. Note that the interactions might be more nuanced if we had been able to test the effects of different haplotypes.

### 4.3 Health parameters

In response to infections by a bacterium and multiple apicomplexa (and probably by other nonidentified pathogens), in addition to the environmental stress caused by captivity and by being repeatedly handled the number of WBC increased rapidly to day 7 PI. This increase in number of WBC differed between experimental and control birds indicating an immunological response to the *M. gallisepticum* infection. The increase in the number of WBC also varied between birds that were coinfected with parasites from different genera of haemosporidia indicating that the strength of the immune response varied with the coinfecting haemosporidia genus. The effect of the type of coinfection was especially clear after day 7 PI when the number of WBC started to decrease. The cost of the immune response following infection with *M. gallisepticum* seemed to be higher in birds infected with parasites from a single‐haemosporidia genus only than when both *Plasmodium* spp. and *Leucocytozoon* spp. were present in the same individual. The strength of the immune response could be associated with the increased parasitemia due to the presence of a novel pathogen (*M. gallisepticum*).

Although the hematocrit values observed in all samples were within the normal range, and the initial decrease was probably due to the frequency of bleeding, differences in the increase in Hct values after day 7 PI were observed between treatment groups. Hct Increased more rapidly in experimental than in control birds and also differed between groups that had different coinfections. Knowing that high corticosteroids have a positive physiological effect on the erythropoiesis, we can speculate that experimental birds might have higher levels of corticosteroids due to coinfection.

From the host perspective, also diverse responses to haemosporidian infections have been observed. Thus, house martins *Delichon urbicum* survival was reduced when birds were infected by haemosporidian parasites, but more so when they have a double infection than a single infection (Marzal, Bensch, Reviriego, Balbontin, & De Lope, 2008); similar to this, people coinfected with malaria and dengue presented more severe clinical signs for both diseases than people infected just with one of the pathogens (Epelboin et al., 2012). Similar to these studies we observed a positive feedback between the two pathogens in our system, coinfected hosts showed more severe clinical signs for both *Plasmodium* spp. parasitemia and mycoplasmal conjunctivitis. It is remarkable that some of these negative effects for the host were only observed when there was a 3‐way interaction in hosts infected by two different genera of haemosporidia and the bacteriu *M. Leucocytozoon* spp. parasitemia only increases when *Plasmodium* spp. was present.

Further research will be needed to determine which immunological mechanisms cause these differences, particularly when *Leucocytozoon* spp. is present in the coinfection. As the physiological effects in the double‐haemosporidia‐infected groups were modest for both health measures, we suggest that competition among haemosporidia can ultimately benefit wild hosts under co‐pathogen stress (e.g., *M. gallisepticum*).

## 5 CONCLUSIONS

Our study shows complex interactions resulting from co‐occurring infections in house finches. Whereby some interactions are beneficial to the pathogens (*M. gallisepticum* and *Plasmodium* spp. seem to both benefit in terms of infection intensity and thus transmission probability when co‐occurring), some are antagonistic (interactions between *Leucocytozoo*n spp. and *Plasmodium* spp.), and some are synergetic (effect of *Plasmodium* spp. on *Leucocytozoon* spp.). Interactions between *Plasmodium* spp. and *Leucocytozoo*n spp. in their exoerythrocytic life stages need to be studied in more detail.

## CONFLICT OF INTEREST

None declared.

## AUTHOR CONTRIBUTION

**María Teresa Reinoso‐Pérez**: Conceptualization (equal); Data curation (equal); Formal analysis (equal); Investigation (equal); Methodology (equal); Project administration (equal); Supervision (equal); Validation (equal); Visualization (equal); Writing‐original draft (equal); Writing‐review & editing (equal). **Keila V. Dhondt**: Conceptualization (equal); Formal analysis (equal); Investigation (equal); Methodology (equal); Resources (equal); Validation (equal); Writing‐review & editing (equal). **Agnes V. Sydenstricker**: Formal analysis (equal); Investigation (equal); Validation (equal); Writing‐review & editing (equal). **Dieter Heylen**: Data curation (equal); Formal analysis (equal); Software (lead); Validation (equal); Writing‐review & editing (equal). **André A. Dhondt**: Conceptualization (equal); Data curation (equal); Formal analysis (equal); Funding acquisition (lead); Methodology (equal); Project administration (equal); Resources (equal); Supervision (equal); Validation (equal); Visualization (equal); Writing‐original draft (equal); Writing‐review & editing (equal).

## Data Availability

All data for this manuscript are freely available at Mendeley Data, V1, https://doi.org/10.17632/czkzkpzfx4.1

## APPENDIX 1

### 1.1

Analysis of GEE parameter estimates with empirical standard errors of the main effects in a linear piecewise regression model for repeated measurements of House finch fitting the (A) White blood cell counts (log‐link; poisson‐distributed residuals) and (B) Haematocrit levels (normal‐distributed residuals) following the inoculation of birds with *M. gallisepticum* in the presence of haemosporidia (*Plasmodium* and/or *Leucocytozoon*). Parameter estimates of the reduced model after backward selection (*p *< .05) are given, based on *p*‐values of score statistics of type 3 tests (see main text). Intercepts (*α*0) for each combination of parasites were left in the model. Only significant main effects on the changes with time (*β*’s) and their lower order interactions were left in the model. The reference groups are the MG infected (MG = 1) and *Leucocytozoon* infected (Hemato = 3).

**Table:**

(A) White Blood cell counts: Analysis of GEE parameter estimates							
Parameter	Hemato	MG	Estimate	Error	*Z*	Pr > |*Z*|	
Intercept			8.7187	0.0781	111.59	<.0001	
MG	0		0.1250	0.0979	1.28	.2017	
MG	1		0.0000	0.0000	.	.	
Haemo	1		−0.1377	0.1284	−1.07	.2838	
Haemo	2		−0.2530	0.1210	−2.09	.0366	
Haemo	3		0.0000	0.0000	.	.	
Haemo *MG	1	0	−0.0715	0.1967	−0.36	.7165	*α*_0_
Haemo *MG	1	1	0.0000	0.0000	.	.	
Haemo *MG	2	0	−0.3700	0.2322	−1.59	.1110	
Haemo *MG	2	1	0.0000	0.0000	.	.	
Haemo *MG	3	0	0.0000	0.0000	.	.	
Haemo *MG	3	1	0.0000	0.0000	.	.	
							
Period 1			0.1775	0.0190	9.35	<.0001	
Period 1*MG	0		−0.0451	0.0225	−2.00	.0454	
Period 1*MG	1		0.0000	0.0000	.	.	
Period 1* Haemo	1		−0.0010	0.0275	−0.04	.9700	
Period 1* Haemo	2		−0.0391	0.0216	−1.81	.0699	
Period 1* Haemo	3		0.0000	0.0000	.	.	*β*_0_
Period 1* Haemo *MG	1	0	−0.0297	0.0442	−0.67	.5012	
Period 1* Haemo *MG	1	1	0.0000	0.0000	.	.	
Period 1* Haemo *MG	2	0	0.0745	0.0287	2.60	.0093	
Period 1* Haemo *MG	2	1	0.0000	0.0000	.	.	
Period 1* Haemo *MG	3	0	0.0000	0.0000	.	.	
Period 1* Haemo *MG	3	1	0.0000	0.0000	.	.	
							
Period 2			−0.0308	0.0049	−6.30	<.0001	
Period 2* Haemo	1		0.0138	0.0075	1.83	.0675	*β*_1_
Period 2* Haemo	2		0.0245	0.0070	3.51	.0005	
Period 2* Haemo	3		0.0000	0.0000	.	.	

Period 1 = (1–*x*) × (day) + Q × *x*; Period 2 = *x* × (day−Q).

*x* = 0 (before manipulation); *x* = 1 (after manipulation); Q = day 7.

*α*_0_: intercept, *β*_0_: slope of the profile curve before flexion (day 7 included), *β*_1_: slope of the profile after day 7.

Levels ‘MG’: 0 = non‐infected; 1 = infected.

Levels ‘Haemo’: 1 = Plasmodium; 2 = both; 3 = Leucocytozoon.

**Table:**

(B) Haematocrit: Analysis of GEE parameter estimates							
Parameter	Hemato	MG	Estimate	Error	*Z*	Pr > |*Z*|	
Intercept			54.1980	1.1881	45.62	<.0001	
MG	0		−2.7189	1.5986	−1.70	.0890	
MG	1		0.0000	0.0000	.	.	
Haemo	1		−2.4140	2.3580	−1.02	.3060	
Haemo	2		−1.3776	1.0709	−1.29	.1983	
Haemo	3		0.0000	0.0000	.	.	
Haemo *MG	1	0	2.0599	3.0140	0.68	.4943	*α*_0_
Haemo *MG	1	1	0.0000	0.0000	.	.	
Haemo *MG	2	0	4.6677	1.6023	2.91	.0036	
Haemo *MG	2	1	0.0000	0.0000	.	.	
Haemo *MG	3	0	0.0000	0.0000	.	.	
Haemo *MG	3	1	0.0000	0.0000	.	.	
							
Period 1			−0.4896	0.1125	−4.35	<.0001	*β*_0_
							
Period 2			0.4903	0.0806	6.08	<.0001	
Period 2* MG	0		−0.1995	0.0864	−2.31	.0209	
Period 2* MG	1		0.0000	0.0000	.	.	*β*_1_
Period 2* Haemo	1		−0.1892	0.1158	−1.63	.1022	
Period 2* Haemo	2		−0.2679	0.0728	−3.68	.0002	
Period 2*Haemo	3		0.0000	0.0000	.	.	

Period 1 = (1–*x*) × (day) + Q × *x*; Period 2 = *x* × (day−Q).

*x* = 0 (before manipulation); *x* = 1 (after manipulation); Q = day 7.

*α*_0_: intercept, *β*_0_: slope of the profile curve before flexion (day 7 included), *β*_1_: slope of the profile after day 7.

Levels ‘MG’: 0 = non‐infected; 1 = infected.

Levels ‘Haemo’: 1 = Plasmodium; 2 = both; 3 = Leucocytozoon.
